# Supplementary material for: The Compensatory Response of Photosystem II Photochemistry to Short-Term Insect Herbivory Is Suppressed Under Water Deficit
Source: Insects. 2025 Sep 21;16(9):984. doi: 10.3390/insects16090984 (PMC12470949; doi:10.3390/insects16090984)
Supplement: Supplementary file 1 [file insects-16-00984-s001.zip › insects-3792582-supplementary.pdf]

insects

MDPI

The Compensatory Response of Photosystem II Photochemistry to Short Term Insect Herbivory Is Suppressed Under Water Deficit

Julietta Moustaka [1], Ilektra Sperdouli [2], Stefanos Andreadis [2], Nikoletta Stoikou [2], Kleoniki Giannousi [3], Catherine Dendrinou-Samara [3] and Michael Moustakas [1,*]

[1] Department of Botany, Aristotle University of Thessaloniki, 54124 Thessaloniki, Greece; ioumoustaka@gmail.com
[2] Institute of Plant Breeding and Genetic Resources, Hellenic Agricultural Organisation-Demeter (ELGO-Demeter), 57001 Thessaloniki, Greece; esperdouli@elgo.gr (I.S.); sandreadis@elgo.gr (S.S.A.); stoikounikoletta@gmail.com (N.S.)
[3] Laboratory of Inorganic Chemistry, Department of Chemistry, Aristotle University of Thessaloniki, 54124 Thessaloniki, Greece; klegia@chem.auth.gr (K.G.); samkat@chem.auth.gr (C.D.-S.)
* Correspondence: moustak@bio.auth.gr

Table S1. Definitions of the chlorophyll fluorescence parameters used in the experiments

Parameter	Definition	Calculation
F_o	Minimum chlorophyll *a* fluorescence in the dark-adapted leaf (PSII centers open)	Obtained by applying measuring photon irradiance of 1.2 μmol photons m^{-2} s^{-1}
F_m	Maximum chlorophyll *a* fluorescence in the dark-adapted leaf (PSII centers closed)	Obtained with a saturating pulse (SP) of 6000 μmol photons m^{-2} s^{-1}
F_o'	Minimum chlorophyll *a* fluorescence in the light-adapted leaf	It was computed by the Imaging Win software V2.41a (Heinz Walz GmbH, Effeltrich, Germany) as $Fo' = Fo/(Fv/Fm + Fo/Fm')$
F_m'	Maximum chlorophyll *a* fluorescence in the light-adapted leaf	Measured with saturating pulses (SPs) every 20 s for 5 min after application of the actinic light (AL) of 426 μmol photons m^{-2} s^{-1} or 1000 μmol photons m^{-2} s^{-1}
F_s	Steady-state photosynthesis	Measured after 5 min illumination time before switching off the actinic light (AL) of 426 μmol photons m^{-2} s^{-1} or 1000 μmol photons m^{-2} s^{-1}
Φ_{PSII}	Effective quantum yield of PSII photochemistry	$(Fm' - Fs)/Fm'$
Φ_{NPQ}	Quantum yield of regulated non-photochemical energy loss in PSII	$Fs/Fm' - Fs/Fm$
Φ_{NO}	Quantum yield of non-regulated energy loss in PSII	Fs/Fm
Fv/Fm	Maximum efficiency of PSII photochemistry	$(Fm - Fo)/Fm$
Fv'/Fm'	Efficiency of the open PSII reaction centers	$(Fm' - Fo')/Fm'$
Fv/Fo	Efficiency of the oxygen evolving complex (OEC) on the donor side of PSII	
ETR	Electron transport rate	Φ_{PSII} × PAR × c × abs, where PAR is the photosynthetically active radiation, c is 0.5, and abs is the total light absorption of the leaf taken as 0.84
qp	Photochemical quenching, representing the redox state of quinone A (Q_A), or in other words the fraction of open PSII reaction centers based on the "puddle" model for the photosynthetic unit	$(Fm' - Fs)/(Fm' - Fo')$

NPQ	Non-photochemical quenching reflecting the dissipation of excitation energy as heat	$(Fm - Fm')/Fm'$
EXC	Excess excitation energy	$(1 - q_p) \times Fv'/Fm'$
1-qL	The fraction of closed PSII reaction centres based on the "lake" model for the photosynthetic unit	$1- (q_p \times F_o'/F_s)$
